# 靶向生物膜的分子印迹策略与进展

**DOI:** 10.3724/SP.J.1123.2025.06028

**Published:** 2026-01-08

**Authors:** Xueting YUAN, Liang WANG, Luxi CHEN, Lianghai HU

**Affiliations:** 吉林大学生命科学学院，超分子结构与材料全国重点实验室，超分子化学生物学研究中心，吉林 长春 130023; Center for Supramolecular Chemical Biology，State Key Laboratory of Supramolecular Structure and Materials，School of Life Sciences，Jilin University，Changchun 130023，China

**Keywords:** 生物膜, 分子印迹聚合物, 细胞外囊泡, 生物医学应用, 综述, biomembrane, molecularly imprinted polymer （MIP）, extracellular vesicle （EV）, biomedical applications, review

## Abstract

生物膜作为细胞内外环境之间的选择性屏障和沟通桥梁，在信号转导、能量传递和物质交换中发挥着重要作用。生物膜由脂质、蛋白质、糖类和其他成分组成，是细胞识别和通讯的枢纽。生物膜介导的特异性识别和结合，在疾病的早期诊断、药物靶向递送、环境监测等诸多方面具有潜在的应用价值。分子印迹聚合物（molecularly imprinted polymer，MIP）作为一种人工抗体，具有成本低、稳定性高和可重复使用等优点，已经成为特异性识别和结合生物膜上生物分子的有力工具。本文概述了针对生物膜上脂质、蛋白和糖分子印迹聚合物的最新进展，并深入探讨了MIP在生物医学领域中的应用。MIP作为高效的分子识别工具，能够实现对疾病生物标志物的高灵敏度和选择性检测；作为药物载体，MIP通过识别特定的疾病标志物，实现了精准的药物靶向递送；在细胞成像方面，MIP用于标记细胞表面的各类生物分子，丰富了成像的分子类型；此外，MIP还可构建传感器，用于检测生物样品中的目标分子。同时，文章也总结了当前MIP面临的主要挑战，包括合成步骤的复杂性、模板去除的不完全性、规模化生产的困难、性能的不足以及糖类模板制备的难题。为应对这些挑战，文中展望了使用虚拟模板或替代模板、整合新兴技术等可行性方案，并深入探讨了MIP应用的生物相容性问题以及应用转化的制约因素，旨在为实现MIP的实际转化应用提供合理的策略。

## Abstract (en)

Biomembranes are selective barriers and communication interfaces between intracellular and extracellular environments. They are crucial for signal transduction， energy transfer， and material exchange. Composed of lipids， proteins， glycans， and other components， biomembranes are central platforms for cell recognition and communication. Their specific recognition and binding abilities show great potential in early disease diagnosis， targeted drug delivery， environmental monitoring， and more. Molecularly imprinted polymer （MIP） has emerged as a robust tool for recognizing and binding biomolecules on biomembranes. This article summarizes recent technological advancements in MIP for biomembrane-associated lipids， proteins， and glycans. Lipids are a key part of biomembranes， crucial for maintaining membrane fluidity and stability. In lipid imprinting， lipid bilayers serve as templates. Functional monomers interact with lipid molecules and， upon polymerization， form a polymeric shell on the bilayer. Template removal leaves behind complementary binding sites. This strategy has been exploited to fabricate lipid-imprinted nanoparticles for drug delivery. These nanoparticles selectively recognize lipid components on cell membranes， thereby enabling targeted drug delivery. Proteins constitute another critical class of biomembrane components and execute diverse functions. Protein-imprinted MIPs selectively recognize membrane proteins such as cell-surface receptors， bacterial outer-membrane proteins， and viral capsid proteins. This enables precise identification and binding of specific proteins， which is useful in disease diagnosis and drug development. MIP can detect specific membrane protein biomarkers on cancer cells， allowing for early cancer detection and monitoring. Glycans also play a key role in biomembranes， particularly in cell recognition and immune responses. Carbohydrate-imprinted MIPs recognize specific glycan structures for use in disease diagnosis and therapy. Cancer cells have different glycan structures on their membranes compared to normal cells. These abnormal glycans serve as biomarkers for early cancer detection and monitoring. The article also emphasizes the potential of MIP in various applications. In disease diagnosis， MIP can develop biosensors for fast and accurate detection of disease biomarkers， enabling early treatment. In drug delivery， MIP can create targeted systems that deliver drugs directly to diseased cells， minimizing off-target effects and enhancing therapeutic efficacy. In cell imaging， MIP can specifically label cells or biomolecules， providing detailed images of cellular processes and aiding in understanding disease mechanisms. In biosensing， MIP can serve as efficient recognition elements to construct biosensors for detecting specific biomarkers in biological samples. The specific binding sites formed by molecular imprinting technology enable MIP-based biosensors to detect target molecules with high sensitivity and selectivity， providing a powerful tool for early disease diagnosis and real-time monitoring. However， the development of MIP still faces challenges， including complex synthetic procedures， incomplete template removal， limited scalability， and the need for performance optimization. The synthesis process is complex， requiring precise control of parameters like functional monomers， cross-linkers， and initiators. Incomplete template removal compromises binding affinity and selectivity. Scaling-up while maintaining batch-to-batch reproducibility is challenging， and the binding capacity， selectivity， and stability of MIPs must be optimized for each application. For glycan imprinting， monosaccharide templates have low specificity， glycan chain templates are difficult to synthesize and purify， and there is a lack of efficient *O*-glycan cleavage tools. To break through the bottleneck， the study improves template removal efficiency， synthetic optimization and structural controllability through controllable free radical/photo-initiated polymerization， magnetic material integration， microfluidics， artificial intelligence， machine learning and 3D printing； meanwhile， virtual templates， glycopeptide substitution templates， and solid-phase imprinting strategies are used to solve the scarcity of glycan templates. In addition， optimization of biocompatible materials， surface modification to reduce toxicity， and combination with computer simulation and automated preparation can accelerate clinical translation. In conclusion， MIP technology is highly promising for biomembrane research with broad applications in disease diagnosis， drug delivery， cell imaging， and extracellular vesicle enrichment. With continued development and optimization， MIP are expected to play an increasingly important role in biomedical and life sciences， offering more powerful tools and solutions for disease diagnosis， treatment， and monitoring.

生物膜作为分布于生物体内外并具有特定功能的薄膜结构，广泛覆盖于所有细胞、微生物和细胞外囊泡（extracellular vesicle，EV）的表面。真核细胞和细菌均具有磷脂双分子层为骨架的质膜。真核细胞质膜的磷脂种类繁多，蛋白质功能多样，糖类主要以糖蛋白或糖脂形式分布在质膜外表面。细菌质膜的磷脂组成相对简单，主要组成是酶和运输蛋白，通常不含糖蛋白，革兰氏阴性菌质膜外层含有脂多糖（lipopolysaccharide，LPS）。病毒不具备细胞结构，但拥有遗传物质和蛋白质衣壳，具备繁殖进化能力和应激反应机制等，是一类独特的生命体。生物膜不仅在调节细胞通讯中起着至关重要的作用，还参与了复杂的生理过程，包括细胞增殖、分化、分泌、迁移、侵袭和吞噬等，并且生物膜还具有维持细胞及胞内活动协调一致、控制物质运输、同时为多种生理活动提供反应场所等其他功能，对生命活动起着重要的支撑作用。

生物膜由脂质、蛋白质和糖类三大类化学成分构成。在结构层面，脂质构成了生物膜双层结构的基本骨架，其中磷脂在脂质成分中占主导地位^［1］^。磷脂分子呈现独特的两亲结构，其亲水头部和疏水尾部有序排列，然后自发组装成双层结构。这种结构不仅赋予生物膜稳定性，而且保证了其适当的流动性，从而为多种生化反应的发生提供了基础平台。脂质代谢紊乱与某些疾病的发生发展密切相关，研究表明神经酰胺的升高与Ⅱ型糖尿病、癌症、阿尔茨海默症等疾病的发生发展相关^［2，3］^。除脂质外，蛋白质、糖类等其他非脂质元素也是生物膜的重要组成成分，它们共同决定了膜生物活性的特异性和广泛性。蛋白质在生物膜中的分布表现出显著的异质性。有些蛋白质以镶嵌的方式嵌入膜中，有些则以吸附的形式附着在膜表面。蛋白质的功能非常多样，根据其结构和位置的不同，可细分为通道蛋白、载体蛋白和受体蛋白等不同的亚型。它们分别在物质的跨膜运输和细胞间的信号传导中发挥关键作用，是维持细胞内外物质和信息交换的核心枢纽。糖类，在生物膜中主要以糖链的形式存在，通过共价键与膜蛋白或膜脂结合形成糖蛋白或糖脂^［4］^，共同构建了一个复杂而精密的生物识别系统，深度参与细胞识别、免疫应答等多种生理机制。细胞膜和EV上的糖类在疾病诊断中起着重要的作用。如膀胱癌患者的尿液EV表现出岩藻糖基化减少和唾液酸化水平增加，为开发基于EV的N-糖组无创诊断标志物建立了基础^［5］^。

生物膜的特异性识别策略在疾病的早期诊断^［6］^、药物靶向递送^［7］^、环境污染物监测^［8］^等多方面具有广阔的应用前景。目前，基于抗体是最常见的识别方法，然而抗体制备复杂昂贵，并且稳定性差^［9］^。核酸适配体作为一种新兴的识别分子，其合成和修饰相对困难，大规模生产成本高，限制了其广泛应用。而基于生物膜的电荷、疏水性等理化性质的识别方法，虽然操作简单，但特异性相对较低，容易得到假阳性或假阴性结果^［10］^。分子印迹聚合物（molecularly imprinted polymer，MIP）是一种可以根据目标分子预先设计和制备的纳米材料，被称为“人工抗体”，MIP具有低成本、高稳定性、普遍适用性和可设计性的优点^［11］^。目前已经使用MIP针对脂质^［12］^、肽^［13］^、蛋白质^［14］^和其他生物分子进行了大量的研究，其特异性识别目标也扩展到细胞^［15］^和微生物^［16］^。分子印迹技术可分为本体印迹、表面印迹、表位印迹，以印迹蛋白质为例，本体印迹将完整蛋白质作为模板，使用功能单体与交联剂形成具有与模板蛋白三维结构互补的空腔，但模板不易去除；表面印迹重点在于蛋白质在MIP表面被特异性识别和结合，模板易洗脱和再次结合；表位印迹则以蛋白质表位肽段作为模板，依靠形成与表位互补的空腔实现特异性识别，模板易制备且特异性高。

目前，已有大量综述介绍MIPs在疾病诊断^［17］^、药物递送^［18］^、样品分离^［19，20］^、病原体检测^［21］^等多方面的应用，但大多聚焦于蛋白质印迹聚合物的应用或者细胞、微生物识别方面的应用，缺乏针对生物膜分子的印迹策略与应用的较为全面的总结。本综述从生物膜组成分类出发，系统介绍针对特定生物分子的印迹策略，概述靶向生物膜分子MIPs的应用进展，并深入分析目前分子印迹技术面临的挑战，提出可行性改进方案。

## 1 生物膜组分的分子印迹策略

### 1.1 脂质

脂质是生物膜的重要组成部分，在维持生物膜的流动性、稳定性等方面起着关键作用。在脂质印迹过程中，脂质体作为一种理想的模板，可以模拟生物膜的结构和性质。通过聚合反应，功能单体在脂质体周围形成聚合物壳层，去除模板后，形成与脂质分子的形状和化学性质互补的印迹空腔。在药物递送领域，脂质印迹纳米载体能够特异性识别细胞膜的脂质成分，从而实现精准靶向给药，提高治疗效果，降低副作用^［22，23］^。生物膜的主要结构脂质是甘油磷脂，包括磷脂酰胆碱（PC）、磷脂酰乙醇胺（PE）、磷脂酰丝氨酸（PS）、磷脂酰肌醇（PI）和磷脂酸（PA），不同类型的脂质发挥着不同的生物学功能。如PS是细胞膜的关键成分之一，通常分布在细胞膜的内叶，当细胞发生凋亡时，PS会翻转到细胞膜表面，暴露在细胞的外环境中，同时也是病毒通过凋亡模拟物介导进入细胞的途径^［24］^。而鞘磷脂（SM）作为PS的结构类似物，由磷酸胆碱头基、鞘氨醇和脂肪酸组成。与PS的特定位置不同，SM广泛存在于细胞膜的外质层中^［25］^，它们的疏水骨架是神经酰胺。对于另一种主要鞘脂——鞘糖脂，还包含基于葡萄糖神经酰胺的单糖、双糖或多糖，有时也含有半乳糖神经酰胺^［26］^。针对生物膜脂质，Zhou等^［27］^发展了一种新的分子印迹策略，是将反相微乳液体系用于磷脂分子的亲水头部基团的印迹，在反相微乳液体系中，利用磷脂的两亲性，使其极性头部基团和疏水性脂肪酸尾定向排列在油水界面，实现了磷脂亲水表位的印迹（图1）。磷脂酰丝氨酸为模板的分子印迹聚合物（PS-MIP）可用于凋亡细胞的选择性标记和尿液EV的分离富集，在靶向药物递送和生物标志物发现领域的研究有着重要的应用价值。在此基础上，Chen等^［28］^进一步利用二氧化钛（TiO_2_）对磷酸基团的亲和力，将其用作内核来定向磷酸基团，促进磷脂亲水性极性头部在油水界面的有序排列，提高结合容量。利用该方法探究了多种磷脂作为模板分子，例如PS、SM、PE和PC，以验证分子印迹的特异性和识别能力。制备的MIP在选择性识别质膜方面具有巨大潜力，为不同磷脂组成的EV亚型的选择性富集提供了一种创新策略。

**图1:**
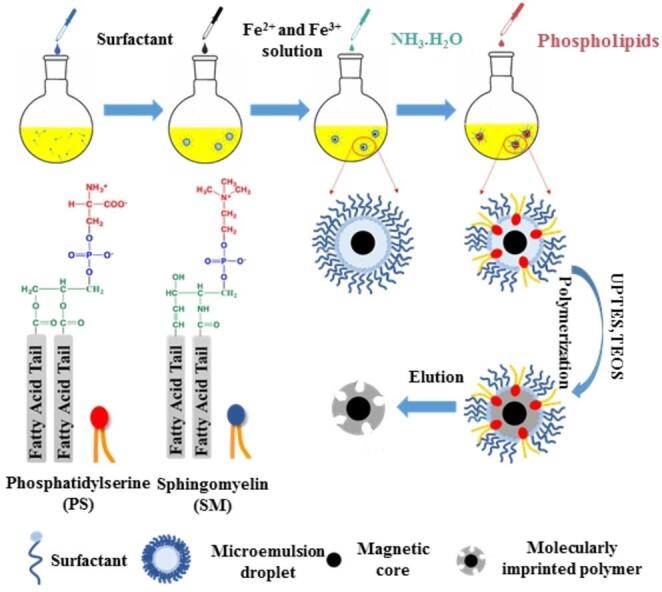
靶向磷脂分子的反相微乳液定向分子印迹策略示意图^［27］^

制备磷脂分子印迹聚合物除了靶向膜磷脂之外，还能利用磷脂与内毒素结构相似的特点，特异性去除内毒素。磷脂酸是一种磷单酯，是甘油磷脂的常见基序，因其含有磷酸单酯和两条脂肪酸链，在结构上与内毒素的脂质A基序有相似之处。Sulc等^［29］^利用优化的单体组合，将磷脂酸作为内毒素的虚拟模板进行印迹。在聚合过程中使用合成的三元复合物对脂质二聚体进行印迹。最终合成的两种MIP能够将内毒素去除至远低于公认阈值的水平，适用于药物生产过程中产品纯化。脂质可以直接作为分子印迹的模板，但由于脂质常与蛋白质等分子相互作用，其原位实时定量仍具有一定困难，亟须开发新型脂质原位实时定量的检测方法。鞘氨醇-1-磷酸（S1P）是一种具有广泛生物活性的鞘脂，与G蛋白偶联受体信号传导密切相关，但S1P含量低且缺乏可靠的检测方法。为此，Li等^［30］^针对S1P和S1P受体调节剂芬戈莫德磷酸盐（FP），开发了一种基于核壳分子印迹聚合物的荧光传感器。该研究采用FP四丁基铵或二棕榈酰磷脂酸作为模板，结合具有捕获磷酸阴离子并传递信号双重作用的功能单体进行制备。合成的MIP能够在稀释血清样本进行高效、高特异性检测，并且灵敏度高。胆固醇是所有动物血液血浆中的主要结构成分，是必需脂质，主要功能是调节膜的流动性、柔韧性和通透性^［31］^。Anirudhan等^［32］^利用分子印迹与电化学传感技术结合，制备胆固醇特异性电化学传感器。通过滴涂法将硅化氧化石墨烯接枝共聚到化学改性纳米纤维素上，对玻璃碳电极进行改性。利用循环伏安法和差分脉冲伏安法，通过铁-铁氧化还原反应来计算测试介质中胆固醇的含量。该胆固醇传感器具有线性范围宽、检出限低、重现性和稳定性好等优点，能从血液样本中分析胆固醇含量，与已建立的临床诊断方法具有良好的相关性。靶向脂质分子的MIP为疾病诊断、药物递送等研究提供了重要的分子工具。

### 1.2 蛋白质

蛋白质作为重要的生物大分子之一，具有种类繁多，功能多样的特点。自从20世纪80年代Glad等^［33］^首次合成用于识别蛋白质的MIP以来，蛋白质作为印迹模板的研究广泛开展。细胞膜蛋白是精准靶向细胞的理想靶标，某些膜蛋白可作为细胞膜上的疾病标志物，如膜蛋白p32受体^［34］^、表皮生长因子受体（EGFR）^［35］^、人成纤维细胞生长因子诱导型14^［36］^、甲型肝炎病毒细胞受体1^［37］^等。除此之外，肿瘤细胞表面某些高表达蛋白能帮助肿瘤细胞逃逸，使用MIP通过阻断对应的信号通路，可以实现癌症免疫治疗。如程序性死亡受体配体1（PD-L1）能与T细胞表面的程序性死亡受体1（PD-1）结合，抑制T细胞活性，从而帮助肿瘤细胞逃避免疫系统攻击。Lu等^［38］^以PD-L1的N端表位为模板，使用硼酸亲和定向表位印迹方法合成了一种针对癌细胞的分子印迹溶酶体纳米降解剂（MILND）（图2）。MILND能靶向PD-L1过表达的肿瘤细胞，促进细胞摄取，随后转运到溶酶体，实现对PD-L1的有效降解，阻断PD-1/PD-L1的信号通路，最终达到持久的抗肿瘤效果。

**图2:**
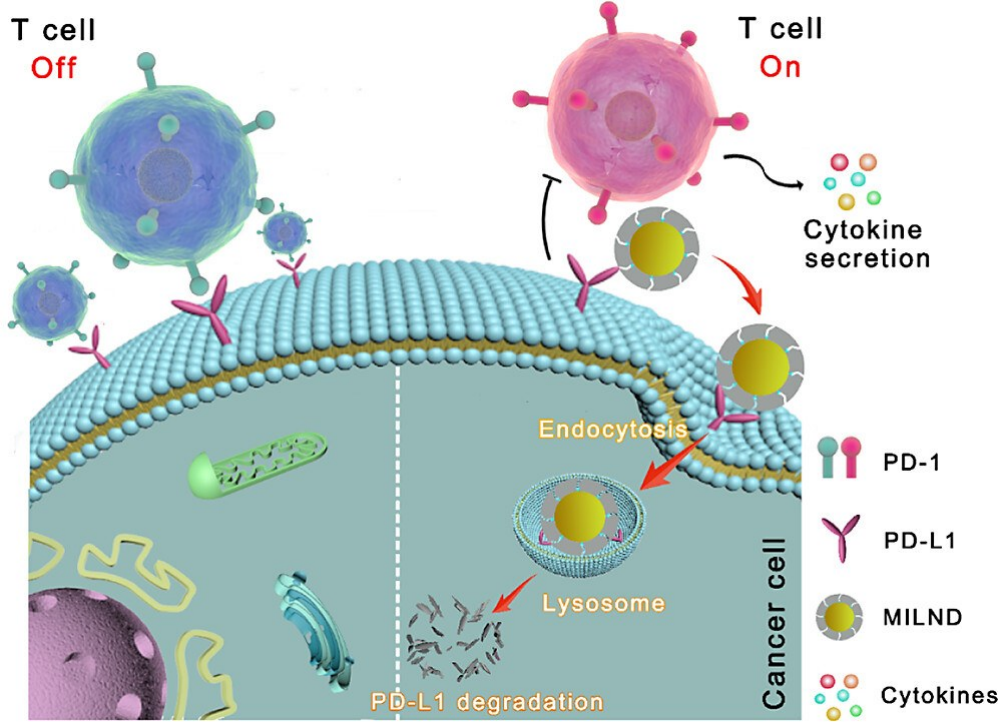
通过MILND逆转PD-L1介导的免疫抑制来增强癌症免疫治疗的示意图^［38］^

外膜蛋白是革兰氏阴性菌外膜的主要成分。作为物质交换的重要入口，在代谢物转运、信号传递和膜发生中发挥重要作用。利用MIP识别特定的细菌蛋白，可用于疫苗开发、疾病诊断和开发新的药物靶点。Gupta等^［39］^通过计算方法从脑膜炎奈瑟菌的外膜蛋白Por B中确定了候选T细胞表位，并成功合成了分子印迹聚合物-石英晶体微天平作为脑膜炎奈瑟菌的诊断工具，随后又针对脑膜炎奈瑟氏球菌铁结合蛋白的肽序列进行印迹，合成的MIP可以特异性识别脑膜炎患者血液样品中的目标蛋白^［40］^。

病毒衣壳蛋白是病毒衣壳的核心结构蛋白，具有保护遗传物质、识别宿主细胞并入侵、促进病毒颗粒组装和释放等功能，在开发疫苗、抗病毒药物等方面具有潜在的研究价值。McClements等^［41］^使用新型冠状病毒的蛋白表位作为模板制备纳米芯片，其亲和力与抗体相当。Xu等^［42］^通过固相合成制备水溶性MIP，靶向包膜蛋白41的表位肽SWSNKS（3S），可在体外阻断3S的功能进而阻断针对CD4+T细胞的级联反应，从而防止CD4+T细胞的损伤并恢复免疫保护。Zhang等^［43］^将刺突蛋白的受体结合域与其表位进行印迹，发现表位印迹聚合物具有成本低、减少非特异性结合的特点，在病毒检测中具有优势。Kaur等^［44］^则利用诺如病毒衣壳蛋白P1结构域暴露的表位序列作为模板合成了分子印迹纳米颗粒（nanoMIP），该纳米粒子可以特异性识别诺如病毒。通过与人工智能的有机结合，可开发新型的多单体组合筛选方法，Rajpal等^［45］^利用数字模拟以严重急性呼吸综合征冠状病毒2（SARS-CoV-2）的表位为例，从常用的硅烷化单体中筛选出与目标表位结合最紧密的单体组合。通过多单体同时对接策略，分析了不同单体与表位的结合亲和力和相互作用点，最终确定了最适合用于MIP合成的单体组合，不仅提高了印迹因子（IF），还减少了实验试错的次数，这种策略适用于大多数的表位模板。

### 1.3 糖类

鉴于糖类在细胞膜和EV上的重要生物学功能及其在疾病诊断中的潜在应用，针对糖类的MIP具有重要意义。利用分子印迹构建的糖基印迹聚合物可以有效地从复杂的生物样品中分离和富集特定分子，并能特异性识别和检测细胞膜和EV上异常糖类的细微变化，为疾病的早期准确诊断和治疗监测提供创新的技术手段^［46］^。

#### 1.3.1 单糖

单糖作为细胞膜上糖链合成的基本单元，参与蛋白质和脂质的糖基化修饰，进而影响细胞膜蛋白的结构及运输、识别等功能。如在肿瘤细胞中，唾液酸（SA）的表达显著升高，掩盖了肿瘤细胞表面的部分抗原决定簇，使免疫细胞难以识别。Lu等^［47］^报道了基于硼酸亲和力的可控定向表面印迹方法，用3-氨基丙基三乙氧基硅烷（APTES）对RNase A@BS-NPs进行氨基化修饰，为硼酸功能化提供活性基团；以四乙氧基硅烷（TEOS）和双-［3-（三乙氧基硅）丙基］-二硫化物（BTEPDS）作为交联剂，制备了SA分子印迹的SiO_2_纳米粒子，用于靶向蛋白递送和癌症治疗。Ma等^［48］^制备了一种SA印迹的热响应性水凝胶层，在37 ℃时具有强SA结合能力，而在较低温度时结合能力较弱，可用于选择性捕获和释放癌细胞。此外，甘露糖（Man）是许多高突变病毒糖盾的保守结构特征。Li等^［49］^制备了一种印迹Man的nanoMIP，能特异性结合高甘露糖糖苷，靶向SARS-Cov-2病毒的糖盾，阻断病毒进入宿主细胞。

#### 1.3.2 糖链

糖链可与蛋白或脂质连接，参与细胞膜上的细胞识别和信号转导，既可用作EV上疾病诊断和监测的标记，又能通过提高对靶细胞的亲和力来提高靶向性和效率，有助于药物递送^［50］^。人类免疫缺陷病毒1型（HIV-1）的包膜糖蛋白（Env）具有大量N-糖基化位点，这些位点充当保护层，使蛋白质免受免疫监视^［51］^。N-糖链在Env糖蛋白的三维排列中表现出显著的易接近性^［52］^。因此，糖链的保守结构特征可能成为实现HIV-1感染抑制的突破口^［53］^。据此，Zhou等^［54］^基于硼酸亲和的表面印迹技术制备了一种糖基印迹纳米颗粒（GINPs），能与分化决定簇抗原4（CD4）竞争性结合Env蛋白，通过空间位阻效应阻止病毒识别靶细胞（图3）。由于HIV-1的Env糖基的保守性，制备的GINPs可以广泛抑制不同HIV-1毒株的感染，包括天然抗体难以中和的二级毒株。

**图3:**
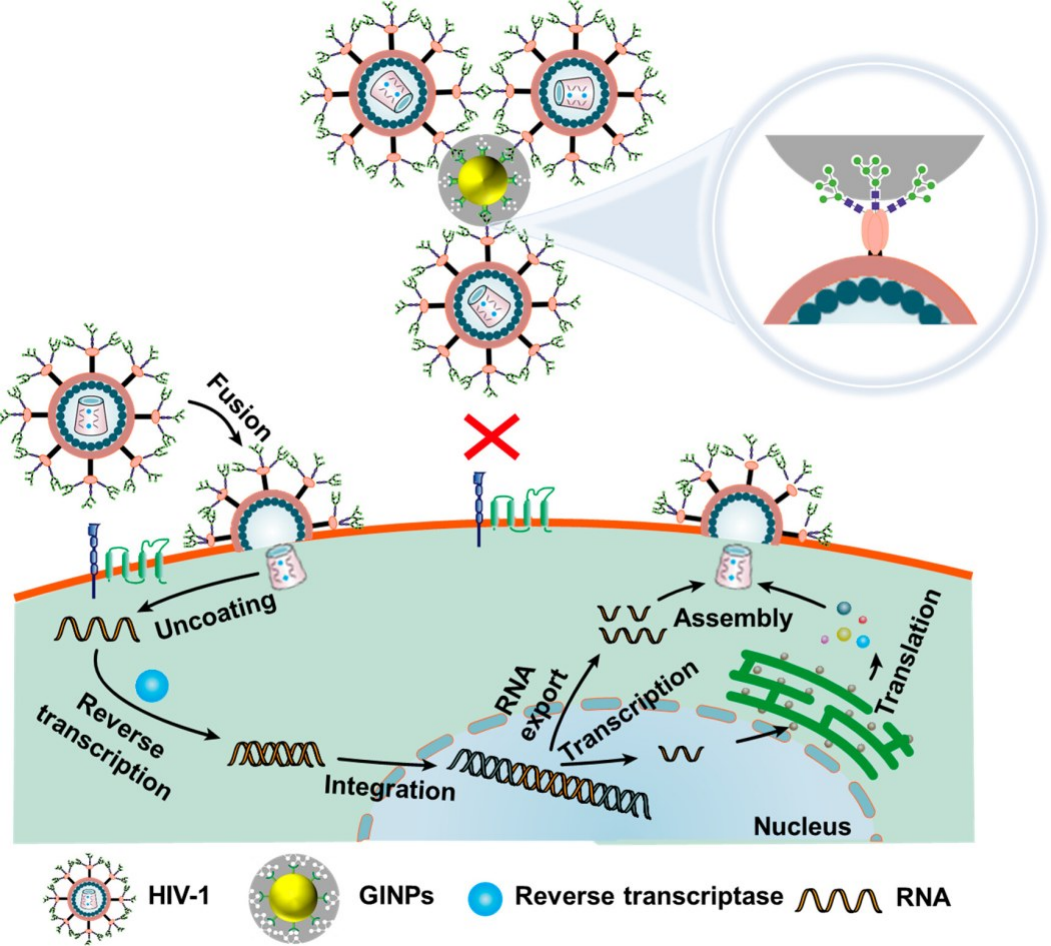
GINPs通过阻断包膜糖链抑制HIV-1感染的示意图^［54］^

#### 1.3.3 糖蛋白

糖蛋白作为细胞膜EV表面糖类的主要存在形式，在细胞间信号转导、免疫反应和各种生理活动中起着重要作用，其异常表达与肿瘤细胞密切相关^［55］^。肽和糖链作为糖蛋白的结构成分，通常用作糖蛋白分子印迹的模板。Guan等^［56］^提出了一种创新性的分子印迹纳米信标（molecularly imprinted nanobeacons，MINBs）策略，选用非转移性黑色素瘤糖蛋白B（glycoprotein nonmetastatic B，GPNMB）的N端表位为模板，并在合成的nanoMIP表面修饰上半抗原荧光素。合成的MINBs能通过特异性结合GPNMB来标记三阴性乳腺癌细胞，从而为半抗原相关的抗体提供精确导航，最终有效杀死三阴性乳腺癌细胞。Zhang等^［57］^以前列腺特异性膜抗胞外顶端结构域的线性表位为模板，合成携带荧光标记的纳米凝胶，可在肿瘤部位特异性富集，从而通过影像学技术对前列腺肿瘤进行精确定位和成像。此外，Jiang等^［58］^利用细胞外表面特异性三糖Neu5Ac-*α*-（2，6）-Gal-*β*-（1-4）-GlcNAc（6′-SLN）为模板，3-氨基苯硼酸（3-aminophenylboronic acid，m-APBA）为功能单体，成功制备糖链印迹聚合物。随后使用这种聚合物构建了一种电化学发光传感器，可以特异性地检测与癌症相关的EV。通过分别以肽和糖链为模板的两种MIP对目标糖蛋白的双重识别，提高了检测特异性，实现了对癌胚抗原的超灵敏检测。Zhou等^［59］^提出了一种基于正反交双分子印迹聚合物的血浆免疫夹心法。使用表位印记的金纳米颗粒识别肽表位，使用具有拉曼活性的银纳米颗粒作为标签识别糖链。这种正反交双识别具有速度快、样品需求少、稳定性高、性价比好等优点，在临床诊断等领域具有广阔的应用前景。

#### 1.3.4 脂多糖印迹

LPS作为革兰氏阴性菌细胞壁的组成部分^［60］^，由O-抗原、核心多糖、脂质A三部分组成，可与免疫细胞的表面受体结合，强烈宿主免疫反应，引发炎症，在抵抗细菌感染中发挥重要作用。同时，它还在细菌生物膜的形成中起着关键作用，增强细菌的抵抗力和生存能力^［61］^，并通过诱导细胞凋亡、改变代谢状态和影响信号通路等多种方式直接影响宿主细胞的功能。

LPS的结构在不同种类细菌间差异很大，特别是O-抗原，由重复的低聚糖单元组成的糖链，具有种属特异性，并在血清型分化中起关键作用^［62］^。LPS独特的结构特征增强了印迹的特异性，能够精确识别目标细菌，同时最大限度地减少与非目标细菌的交叉反应。Lee等^［63］^使用聚多巴胺对沙门氏菌的LPS进行印迹，制备传感器。LPS的O-抗原含有D-鼠李糖残基，这些残基引入了顺式二醇基团，能与硼酸功能化的MIP发生强相互作用。因此，沙门氏菌LPS印迹的MIP在采用电化学方法检测食源性病原体时表现出高灵敏度和选择性。Kinoshita等^［64］^印迹大肠杆菌的O157-抗原，在包覆金纳米粒子（gold nanoparticles，Au NPs）的*N*-异丙基丙烯酰胺（*N*-isopropylacrylamide，NIPAm）共聚物上形成与模板形状互补的空腔。利用NIPAm的生物相容性和Au NPs的光散射性，对O157-抗原实现特异性识别和光学标记。MIP与光热疗法结合，可实现对细菌靶向和杀灭。Zhang等^［65］^以铜绿假单胞菌（*P. aeruginosa*）的LPS为模板，将LPS靶向的硼酸亲和性MIP与光热灭活相结合，有效灭活了铜绿假单胞菌。LPS印迹聚合物除了能特异性识别细菌，也能用于捕获外膜囊泡（OMVs）。Li等^［66］^以LPS作为模板分子，在TiO_2_纳米粒子表面包覆SiO_2_，形成含有印迹空腔的磁性纳米粒子。通过控制涂层的厚度和均匀性，形成了与LPS形状互补的印迹空腔（图4）。所制备的MIP可在40 min内从100 μL介质中高效分离OMVs，回收率超过95%，同时具有良好的重复利用性。基于OMV的生物标志物筛选研究为细菌感染性疾病的快速诊断提供了新的策略。

**图4:**
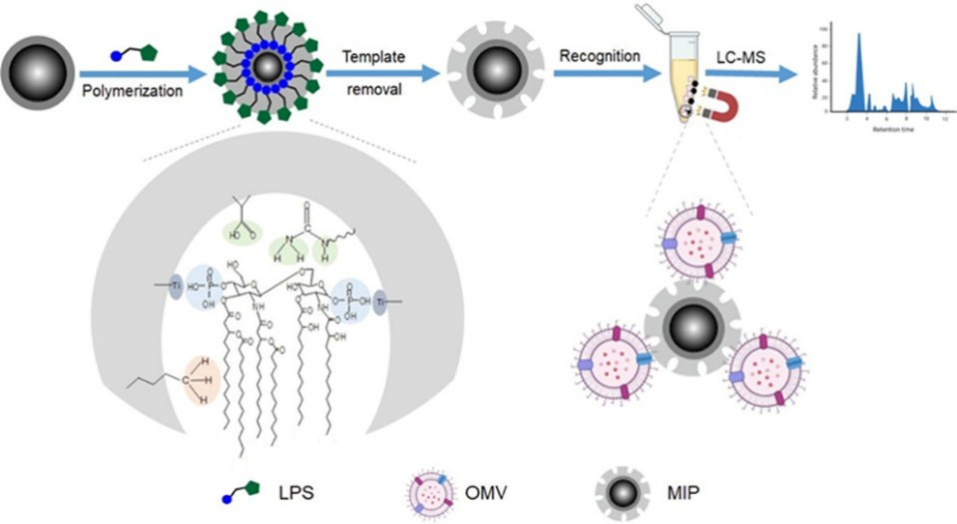
LPS分子印迹聚合物合成过程及蛋白质组学应用^［66］^

## 2 分子印迹聚合物的生物医学应用

### 2.1 疾病诊断

生物标志物是疾病发生和发展的关键指标，为早期诊断和治疗监测提供重要信息。MIP可被设计用于特异性识别和结合蛋白质、核酸和代谢物等生物标志物。EV作为细胞间通讯的载体，广泛存在于多种体液中，正逐渐成为一种富含代谢物、蛋白质和核酸的疾病诊断标志物。EV中的蛋白质和RNA可以作为早期诊断和疾病监测的生物标志物^［67］^，其携带的各种信号分子能传递给靶细胞以调节生理功能^［68］^。同时，作为一种天然的药物传递载体，可以将药物传递到特定的靶细胞，提高药物的疗效，减少副作用^［69］^。然而，由于EV含量低且与蛋白质复合物结构相似，高效分离EV是生物医学应用中最重要的问题之一。MIP已经成为EV富集和分析的有力工具，在特异性、效率和多功能性方面具有显著优势。Takeuchi等^［70］^通过动态成型制备了能特异性识别小细胞外囊泡（small extracellular vesicle，sEV）的信号纳米腔，并将其与抗体偶联，实现了对sEV的高灵敏度、高选择性检测，为癌症的早期诊断提供了一种新型无创方法。

由于线粒体和ATP生成机制的存在，在从细胞质裂解物中分离EV时，质膜的对称性会受到破坏，导致EV表面暴露出PS、PE等磷脂^［71］^。因此，EV膜中磷脂的分布特征是其识别和分离的关键基础。胰腺癌是胰腺常见的恶性肿瘤，发病隐匿且早期诊断困难。Cheng等^［72］^通过PS-MIP富集和质谱分析对胰腺癌尿液EV进行蛋白质组学表征。胰腺癌患者与健康对照组相比，蛋白质组谱中最显著的变化是溶质载体家族9成员3调节器1、精子相关抗原9和铁蛋白轻链的过表达，这些蛋白质可能在诊断和预后评估中发挥重要作用。除了检测尿液中EV，PS-MIP在富集血浆EV方面仍有极高特异性^［73］^，通过蛋白质组学分析筛选了胰腺癌诊断和预后评估的潜在生物标志物，并可能为胰腺癌的进一步临床研究提供支持。基于EV的脂质组学为小分子代谢类标志物的筛选提供了新的途径，Chen等^［28］^开发了靶向磷脂的新型表位印迹策略，实现了对PS、SM、PC、PE的特异性识别，利用MIP分离EV的脂质组学研究筛选了肝癌、肝硬化和健康对照组的差异脂质（图5）。同时混合材料显示出更高的富集效率，并且可以抵消不同磷脂来源的EV的异质性，为更全面的液体活检提供了新策略。MIP还可与表面增强拉曼等进行联用，以提高检测的灵敏度，Zhao等^［74］^利用PS-MIP开发了两步靶向检测方法。首先通过磷脂极性位点印迹策略富集EV，然后利用纳米酶联免疫吸附技术实现对EV的高灵敏度检测，将分子印迹技术与拉曼光谱相结合，实现了胰腺癌患者尿液标志物的无创和高灵敏检测，为肿瘤的早期筛查和治疗监测开辟了新途径。

**图5:**
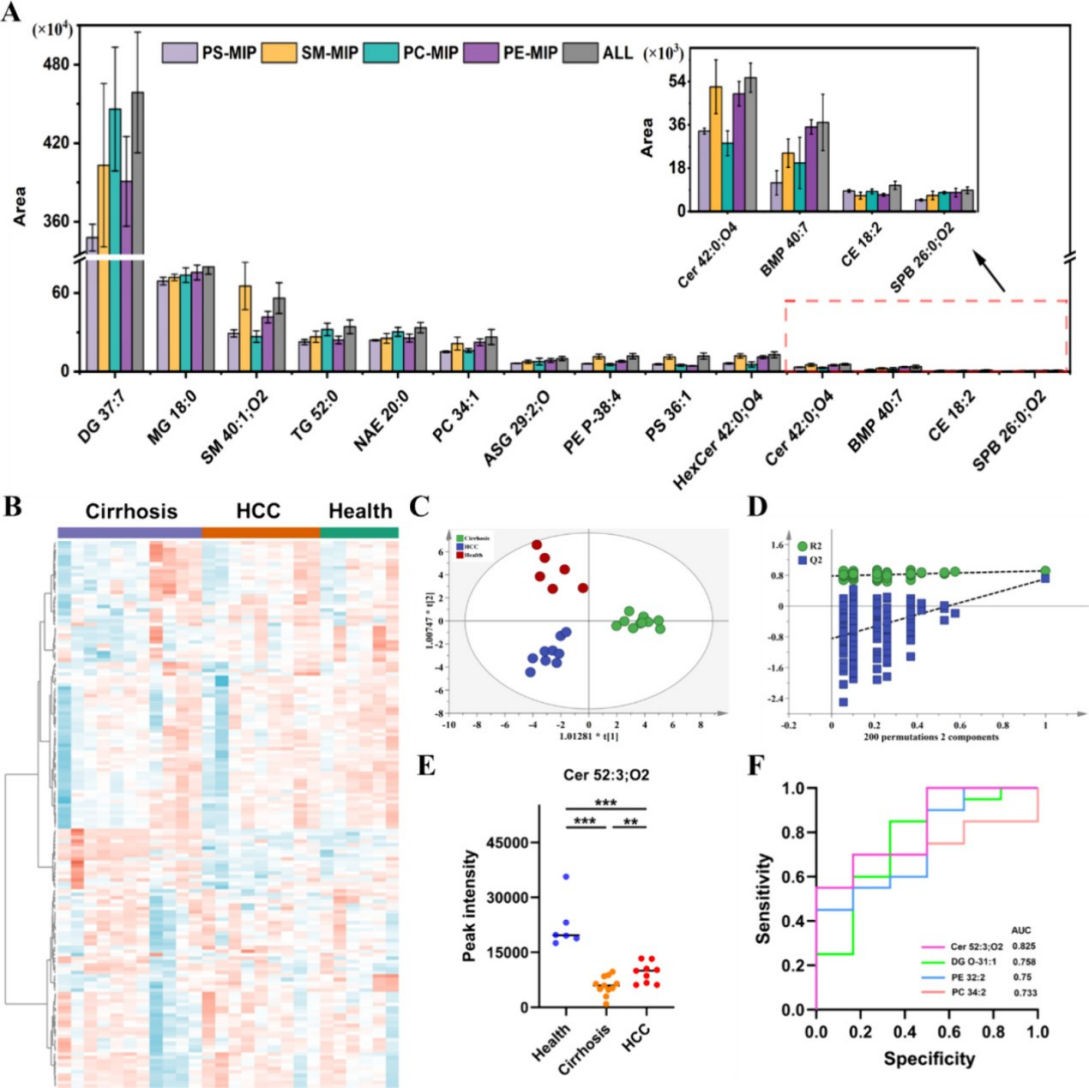
尿液EV的脂质组学分析^［28］^

此外，MIP与比色检测等技术联合，将为临床诊断提供更加经济高效且简便的方法。Zhang等^［75］^使用双分子印迹免疫夹心比色策略，对血清中肺癌标志物α2巨球蛋白实现即时诊断。首先使用玻璃片为基底的分子印迹材料作为“分离抗体”，从复杂样品中快速分离目标蛋白，后利用不对称修饰的分子印迹金纳米酶作为“检测抗体”，特异性识别目标蛋白并催化底物显色，从而可以通过肉眼观察底物的颜色变化来确定α2巨球蛋白的浓度，为即时诊断提供了通用工具，同时为抗体模拟技术、多功能抗体制备提供了新视角。MIP作为人工抗体，是天然抗体的有效替代品（表1），并且由于其成本效益、高特异性、易于合成、高稳定性和可重复使用等特性^［76］^，已被有效地应用于疾病诊断领域。

**表1:** MIP与抗体的特征比较

Characterization	MIP	Antibody
Specificity	moderate specificity	high specificity， accurate identification
Stability	stable at room temperature long-term	susceptible to inactivation
Production cost	low	high
Production time	short	long
Reusability	stable and reusable for many times	poor reusability
Feasibility of large batch production	relatively simple preparation and easy to synthesize at large scale	requires strict process control and quality assurance measures

### 2.2 药物递送

MIP可作为药物载体，通过其独特的分子识别能力，实现对肿瘤细胞的精确靶向，从而提高药物疗效，减少对正常细胞的损伤。此外，MIP还可以提高载药量和稳定性，改善药物的药代动力学性质，为肿瘤治疗提供更有效的手段。EGFR作为许多癌症的生物标志物，其突变或过度表达与许多恶性肿瘤的发生和发展有关。Piletsky等^［35］^利用EGFR肽作为模板印迹，在肽的末端添加半胱氨酸进行固相偶联，通过比较氨基硅烷和碘硅烷的性质，表明使用碘硅烷的MIP对于印迹EGFR表位表现出优异的均匀性、亲和性和特异性。单个分子的印迹通常无法同时实现药物递送和精确靶向，因此研究人员开发了利用肿瘤标志物和抗癌药物的两种表位作为双模板制备的MIP。由于质膜不仅通过其磷脂双分子层维持细胞内微环境，而且还消除细胞膜外的外源性化合物的性质，大多数药物，尤其是高极性的药物，被阻止进入细胞发挥作用。因此，Cheng等^［77］^以质膜上高表达的SM和治疗HIV和慢性乙型肝炎的药物替诺福韦（TFV）为模板分子，制备了一种载药量高的双模板分子印迹聚合物（dual-templated MIP，dt-MIP），可以通过内吞作用被细胞内化，这种机制已广泛用于治疗癌症的纳米药物设计中（图6）。在模拟生理条件下的体外药物释放实验表明，dt-MIPs对TFV的吸附率在50 h内始终保持在80%以上，表现出持续且稳定的缓释性能。通过流式细胞术和共聚焦显微镜的结合，发现dt-MIP具有有效的细胞通透性。此外，基于质谱的细胞内药代动力学研究表明，dt-MIP在30 min内即可将TFV高效递送至细胞内。上述研究表明，开发的dt-MIP有望成为增强药物的细胞透膜性和控释的替代纳米级药物载体。双模板印迹除了用于提高药物递送效率，在免疫检查点阻断疗法中也发挥有效作用。Guan等^［78］^以PD-L1和信号调节蛋白α（SIRPα）的两个表位为模板，设计了一种双特异性分子印迹纳米免疫锁（bsMINIB），可特异性结合肿瘤细胞上的PD-L1和巨噬细胞上的SIRPα。BsMINIB通过阻断PD-L1/PD-1和CD47/SIRPα信号通路来恢复巨噬细胞的吞噬作用，促进了肿瘤相关抗原的呈递，并重新激活了T细胞的免疫作用，最终有效抑制肿瘤生长。

**图6:**
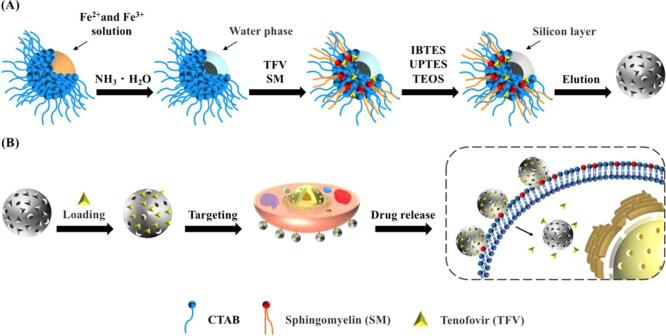
（A） dt-MIP 的合成流程图；（B）通过靶向细胞膜实现 dt-MIP 的药物负载和控释^［77］^

### 2.3 细胞成像与生物传感

MIP作为具有前景的人工受体，可被设计为特异性结合靶标，易于掺入各种纳米材料或染料，并且具有物理和化学稳定性^［79-81］^。因此用于特异性识别并结合细胞膜上分子的多功能nanoMIP已被广泛报道用于靶向细胞成像^［82］^。Panagiotopoulou等^［83］^制备了两种荧光标记的nanoMIP，一种以葡萄糖醛酸为模板，靶向细胞外硫酸软骨素位点；另一种以*N*-乙酰神经氨酸为模板，靶向细胞内SA位点。绿色和红色发光的量子点分别被葡萄糖醛酸和*N*-乙酰神经氨酸功能化，实现了多重细胞成像。这种通用方法，也可以适应细胞表面和内部的其他靶标分子。Shen等^［84］^制备了含硅量子点的表面印迹聚合物。由于硅量子点的细胞毒性低，该MIP可用于细胞内源性谷胱甘肽的荧光成像，在实际样品中进行高分辨率靶向癌细胞成像具有巨大潜力。

生物传感器集成了受体与转换器，能够利用生物识别元件特异性定量或半定量的分析信息。基于MIP的传感器不仅具有灵敏度高、响应速度快、成本低等传统传感器的优势，并且具有可设计性，进一步定制能用于临床检测^［17］^。该传感器利用MIP来模拟酶或抗体对目标分子特异性识别。并利用信号放大技术放大电化学、荧光、比色等信号，有效实现生物样品中低丰度目标的实时检测和定量^［85］^。Ratautaite等^［86］^以SARS-CoV-2-S的刺突糖蛋白为模板合成分子印迹聚吡咯（MIP-Ppy），MIP-PPy层修饰在铂电极表面，通过脉冲安培检测法监测电流变化。SARS-CoV-2蛋白与空腔结合，阻碍电极表面电子转移，导致电流响应下降，从而实现对SARS-CoV-2病毒蛋白的检测。利用具有特定糖链结构的EV表面特征糖蛋白的糖链作为模板分子，通过电聚合制备GIP膜，多步反应合成的传感器可用于EV的高灵敏度和选择性检测。Jiang等使用6′-SLN作为模板分子，通过电聚合形成糖基印迹聚合物，制备了两种类型的电化学传感器：一种用于检测癌症相关的EV^［58］^，另一种用于监测医疗废物中的sEV^［87］^。在前一种传感器中，当EV存在时，GIP膜中的印迹空腔特异性结合EV表面的糖链，核酸适体识别并结合EV表面的特定蛋白质，随后通过电化学发光反应进行检测。这种传感器具有高灵敏度和高选择性，能够快速准确地检测癌症相关的EV，从而为早期癌症诊断和监测提供了强有力的工具。后一种传感器使用m-APBA分子作为信号探针，通过硼酸亲和作用识别糖蛋白的糖链来捕获sEV。m-APBA通过硼酸亲和力固定在sEV表面的糖蛋白上。这种传感器适用于检测复杂样品如医疗废水和尿液中的sEV，对于防止传染病的传播和处理医疗废物中的环境污染物具有重要意义。

## 3 结论与展望

分子印迹聚合物作为一种人工抗体，通过靶向生物膜能发挥疾病诊断、药物递送、细胞成像等多种功能。然而，目前现有的MIP存在一些问题，包括制备步骤复杂、模板难完全去除、规模化生产困难、性能较差等。针对糖类的分子印迹，还存在模板制备困难的问题。单糖模板虽然稳定易得但制备的MIP特异性差，糖链模板化学合成制备困难，主要通过糖苷酶酶解后纯化获得，纯化过程复杂且产率低，同时缺乏能直接从糖蛋白中切割O-聚糖的酶。为了解决这些问题，研究人员已经尝试通过可控自由基聚合^［88］^或光引发聚合^［89］^以提高模板移除效率和可扩展性。并且引入磁性材料，提高MIP在样品分离的效率^［90］^。在此基础上，MIP与新兴技术的整合可以进一步增强MIP性能，如微流控（microfluidics）^［91］^、人工智能（artificial intelligence，AI）、机器学习（machine learning，ML）^［92］^、3D打印等^［93］^。集成有MIP的微流控装置已被开发用于快速有效地分离复杂样品中的微生物。AI驱动的MIP设计可以优化其与特定目标的结合特性。ML已被用于优化MIP的合成条件，预测印迹因子和模拟结合亲和力^［94］^。MIP的传统开发过程通常需要大量反复的试验，包括调整化学组成和交联剂比例。ML通过训练算法，能够快速预测最佳的化学成分和合成条件，从而减少实验的数量和成本。3D打印用于MIP，或能根据计算机辅助设计模型的设计，创建具有复杂和定制结构的印迹空腔，实现印迹位点空间排布的一致性，解决MIP印迹位点异质性问题，提升识别效率。并且3D打印可用于打印支架，为MIP的使用提供便利^［95］^。针对糖类印迹的模板难获取问题，可以采用虚拟模板或替代模板。虚拟模板具备与目标模板相似的尺寸分布和化学性质，制备的MIP具有与模板形状和分子结合特征精确匹配的纳米空腔^［96］^。替代模板为糖肽，印迹时仅印迹替代模板的糖基部分，制备的MIP表现出优异的糖基特异性，且同时适用于N-糖基、O-糖基及单一糖基和多种不同糖基的同时印迹^［97］^。除此之外，采用固相印迹能实现糖类等珍贵模板的重复利用。固相印迹选用表面经模板分子修饰的无孔玻璃珠作为固定化模板相，在印迹过程中多次重复使用了分子模板，同时控制了模板的方向，提高了MIP的特异性^［98］^。

MIP在生物医学的实际应用过程中，还需考虑生物相容性及体内应用风险。某些MIPs如SiO_2_纳米粒子，具有良好的生物相容性和较低的毒性^［99］^，其安全性通过动物模型已得到初步验证，并且已进入Ⅱ期的临床试验，显示出巨大的应用潜力^［100］^。多数用于合成MIP的单体和聚合物基质本身生物相容性较好，同时通过表面改性从而可降低MIP的免疫原性和非特异性吸附，进而提高其生物相容性。然而，部分功能单体（如丙烯酸酯）可能会对细胞膜造成损伤或引发线粒体功能障碍。目前，关于MIP长期毒性和代谢的研究相对较少，亟需根据其使用需求进行深入探究、个性化设计和优化。与抗体相比，MIP在大分子识别效率方面相对较低，只能对稳定构象进行印迹等局限性。这些因素在一定程度上限制了MIP在体内的应用范围和应用转化。为了推动MIP的实际应用转化，可采取以下策略：首先，优先选用生物相容性优越的材料来合成MIP；其次，借助自动化设备精确控制反应条件，从而提升MIP的质量稳定性；最后，利用计算机模拟和建模技术预测和优化MIP的结构与性能，以此指导实验设计和制备过程，既能提高研发效率，又能降低研发成本，进而加速MIP技术在生物医学等领域的应用转化进程。

靶向生物膜的MIP在生物医学方面的多方向应用中显示出巨大的潜力，但在实际应用中还存在诸多的问题，如模板的重复使用和高效去除、材料制备的稳定性和规模化、生物相容性和特异性等。未来通过发展新型的分子印迹技术，将材料的制备进行标准化，降低本身的非特异性吸附，并将MIP与ML、3D打印等先进技术相结合，利用计算机模拟优化合成过程，使用3D打印制备复杂空腔或结构，提高材料合成的智能性和靶向性，推动MIP的创新发展和在更多研究领域的广泛应用。
